# Nuclear envelopathies: a complex LINC between nuclear envelope and pathology

**DOI:** 10.1186/s13023-017-0698-x

**Published:** 2017-08-30

**Authors:** Alexandre Janin, Delphine Bauer, Francesca Ratti, Gilles Millat, Alexandre Méjat

**Affiliations:** 1 University Lyon, Université Claude Bernard Lyon 1, Institut NeuroMyoGène, F-69622 Villeurbanne, France; 2 CNRS UMR 5310, F-69622 Villeurbanne, France; 3 INSERM U1217, F-69622 Villeurbanne, France; 4 Laboratoire de Cardiogénétique Moléculaire, Centre de Biologie et Pathologie Est, Hospices Civils de Lyon, Lyon, France; 5 Nuclear Architecture Team, Institut NeuroMyoGène, CNRS UMR 5310 - INSERM U1217 - Université de Lyon - Université Claude Bernard Lyon 1, Lyon, France; 6 Groupement Hospitalier Est – Centre de Biologie Est – Laboratoire de Cardiogénétique, 59 Boulevard Pinel, 69677 Bron, France

**Keywords:** LINC complex, Nuclear envelope, Lamins a/C, *LMNA*, Sun, Nesprins, *EMD*, LAP2, ZMPSTE24

## Abstract

Since the identification of the first disease causing mutation in the gene coding for emerin, a transmembrane protein of the inner nuclear membrane, hundreds of mutations and variants have been found in genes encoding for nuclear envelope components. These proteins can be part of the inner nuclear membrane (INM), such as emerin or SUN proteins, outer nuclear membrane (ONM), such as Nesprins, or the nuclear lamina, such as lamins A and C. However, they physically interact with each other to insure the nuclear envelope integrity and mediate the interactions of the nuclear envelope with both the genome, on the inner side, and the cytoskeleton, on the outer side. The core of this complex, called LINC (LInker of Nucleoskeleton to Cytoskeleton) is composed of KASH and SUN homology domain proteins. SUN proteins are INM proteins which interact with lamins by their N-terminal domain and with the KASH domain of nesprins located in the ONM by their C-terminal domain.

Although most of these proteins are ubiquitously expressed, their mutations have been associated with a large number of clinically unrelated pathologies affecting specific tissues. Moreover, variants in SUN proteins have been found to modulate the severity of diseases induced by mutations in other LINC components or interactors. For these reasons, the diagnosis and the identification of the molecular explanation of “nuclear envelopathies” is currently challenging.

The aim of this review is to summarize the human diseases caused by mutations in genes coding for INM proteins, nuclear lamina, and ONM proteins, and to discuss their potential physiopathological mechanisms that could explain the large spectrum of observed symptoms.

## Background

Discovered in 1994, the *EMD* gene that codes for emerin, was the first molecular etiology for X-linked Emery-Dreifuss Muscular Dystrophy (EDMD) [1]. At the time, several mutations affecting sarcolemmal proteins had been shown to be responsible for muscular dystrophies. Based on the presence of a hydrophobic helix in its C-terminal domain, it was suggested that emerin could be a membrane protein of the secretory pathway, involved in vesicular transport [1, 2]. However, emerin was unexpectedly found to be embedded in the inner nuclear membrane (INM) [3, 4].

**Fig. 1:**
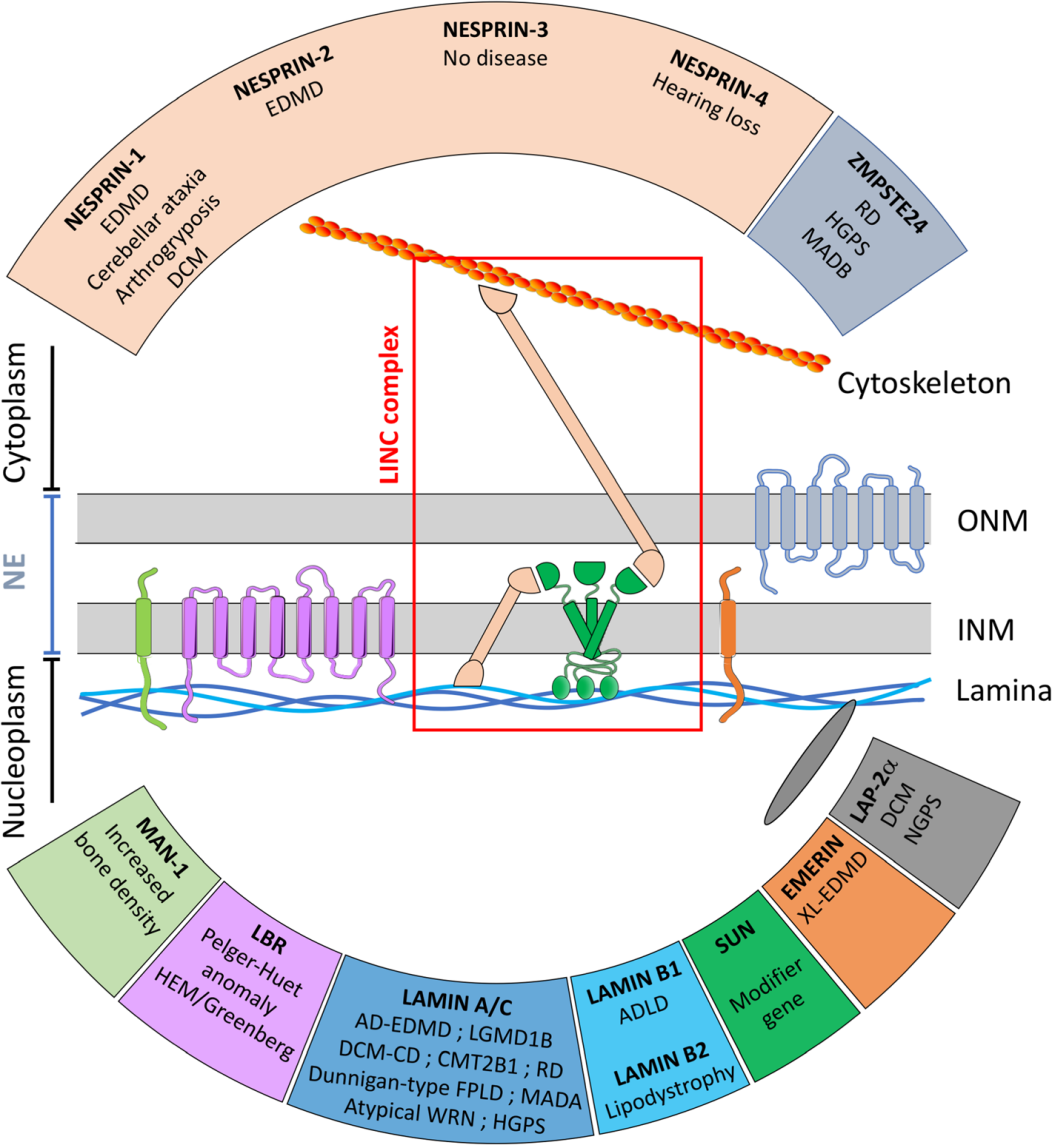
The LINC complex, its interactors, and associated diseases. Schematic representation of the different nuclear envelope components and their interactions. The pathologies associated with mutations in the related genes are indicated in the corresponding boxes. LINC complex components (SUN proteins in green and Nesprins in light brown) are highlighted in the red box. EDMD: Emery-Dreifuss Muscular Dystrophy, RD: Restrictive Dermopathy, HGPS: Hutchinson-Gilford Progeria Syndrome, MADA: Mandibuloacral Dysplasia type A, MADB: Mandibuloacral Dysplasia type B, DCM: Dilated Cardiomyopathy, DCM-CD: Dilated Cardiomyopathy with Conduction Defects, NGPS: Nestor-Guillermo Progeria Syndrome, ADLD: Autosomal Dominant Leukodystrophy, LGMD: Limb-Girdle Muscular Dystrophy, CMT: Charcot-Marie-Tooth, FPLD: Familial Partial Lipodystrophy, WRN: Werner’s Syndrome

Since the discovery of the *EMD* gene, mutations in other genes encoding components of the INM or outer nuclear membranes (ONM), or the nuclear lamina covering the inner side of the nuclear envelope were found to be responsible for several diseases collectively called “nuclear envelopathies” (Fig. 1). Surprisingly, most of these diseases are tissue-specific, affecting the skeletal muscle, heart, peripheral nerves, bone(s) or adipose tissue, whereas they are caused by mutations in ubiquitously expressed proteins. Mutations in *LMNA*, encoding lamins A and C, two main components of the nuclear lamina mediating the interactions with chromatin and gene expression regulators, lead to the initial hypothesis that the mutated nuclear lamina could be responsible for an alteration of the interactions between tissue-specific transcription factors [5]. Since, mutations in nesprins and SUN proteins, transmembrane proteins forming a physical link between the nucleoskeleton and the cytoskeleton (LINC complex) suggest that nuclear envelope disorganization could mechanically lead to nuclear fragility, misresponse to mechano-transduction and/or aberrant signaling events [6–9]. Nowadays, several non-exclusive pathophysiological mechanisms have been proposed, none of them completely explaining the observed defects in patients.

The growing number of proteins identified as interacting with the LINC complex and the diversity of associated diseases are currently a challenge in terms of genetic and clinical diagnosis as several clinically-unrelated diseases could be due to mutations in a common gene, and, conversely, mutations in several genes encoding different components of the nuclear envelope could lead to the same cellular defect and pathology. The situation got even worse with the recent discovery of mutations, or variants, in the gene encoding for LINC component SUN, which were not directly responsible for a disease but, combined to a mutation in a gene encoding for another LINC component, could determine its level of severity.

The aim of this review is to exhaustively summarize our current knowledge of the numerous proteins composing LINC complexes, their interactors and their implication in rare human pathologies.

## Definition of the LINC complex and its interactors

The nuclear envelope is a double membrane isolating the genetic material and separating the inside of the nucleus from the cytoplasm. These two membranes include transmembrane proteins providing a structural support to the nucleus and a physical coupling between the cytoskeleton and the nucleoskeleton. This complex, called the LINC (Linker of the Nucleoskeleton to the Cytoskeleton) complex, is composed of proteins of the outer and the inner nuclear membrane that constitute the core of the LINC complex: KASH (Klarsicht, Anc-A and Syne Homology) and SUN (Sad1 and UNC-84) homology domain proteins [10]. SUN proteins are inner nuclear membrane proteins which interact with nuclear pore complex proteins and lamins via their N terminal domain. Their C-terminal SUN domain is located in the perinuclear space and mediates the interaction with the C-terminal KASH domain of nesprins located in the outer nuclear envelope [11–13] (Fig. 1).

In the following paragraphs, mutations first affecting genes encoding for components of the inner nuclear membrane, then composing the nuclear lamina and finally participating to the outer nuclear membrane will be further developed.

## Inner nuclear membrane (INM) proteins and inherited diseases

### Mutations in the EMD gene

Emery-Dreifuss Muscular Dystrophy (EDMD) is usually described by a triad of three main symptoms. First, early contractures of the elbows flexors, Achilles tendons, and post-cervical muscles lead to rigidity of spine. These symptoms mostly appear during the early adolescence. Then, slowly progressive muscle wasting and weakness (humero-peroneal in the early stages and then more diffuse impairments) and, finally, cardiac disease with conduction defects and arrhythmias will occur [14].

The diagnosis of EDMD is based on clinical findings. Some non-specific biological parameters could orient the diagnosis, such as moderate elevation of Creatine-Kinase (CK) levels in serum or plasma (from 2 to 20 times the upper normal limit), indicating a muscular cell lysis process but, most often, serum CK concentration is normal [15]. The histological examination of muscular sections is another tool: the muscle histopathology usually does not find any specific myopathic or dystrophic disruptions such as variation in muscle fiber size, fibrosis, or necrosis. The most common observed sign is variation in fiber size and increase in internal nuclei. Electron microscopy finds alterations in nuclear architecture: hypercondensed chromatin, nuclear fragmentation or invaginations, intranuclear filaments. Immunodetections of emerin by immunofluorescence or western blot in tissues could also be informative [15]. However, muscular biopsy is rarely performed as it is invasive for the patient and not necessary in case of typical signs. Due to the risk of sudden death [15], it is essential to establish the extent of disease immediately following the initial diagnosis. A cardiac evaluation (ECG, echocardiography, and cardiac Magnetic Resonance Imaging or MRI), a spirometric examination, and the evaluation of the presence of other cardiac risk factors (such as endocrinological abnormalities) are highly recommended [15, 16].

Nevertheless, molecular genetic testing is the main approach to diagnose EDMD confronted with clinical features. Mutations in one gene, *EMD*, are known, since 1994, to be responsible for X-Linked EDMD (also called XL-EDMD or EDMD1). This form has great clinical and genetic heterogeneity. The cardiac symptoms are usually limited to arrhythmias and dilated cardiomyopathies are extremely rare [17] (Fig. 4a). Its prevalence was initially reported in 2002 to range from 1/300,000 to 1/100,000 [18] but, refined in 2009, and estimated between 0.13/100,000 and 0.2/100,000 [19].

This gene encodes for emerin which is located in the inner nuclear membrane and interacts with nuclear lamins. Emerin, which is ubiquitously expressed, is involved in the regulation of gene expression, cell signaling, and nuclear architecture [20]. It is a member of the LEM-domain protein family, that includes LAP2β (Lamin Associated Protein) and MAN1, and which can bind to Barrier-to-Autointegration Factor (BAF). The LEM-domain of emerin, located in the N-terminal part of protein, can adopt a helix-loop-helix fold crucial for its binding to BAF, which is an essential protein involved in post-mitotic nuclear assembly, cell viability, and cell cycle progression. BAF also plays a central role in nuclear envelope reformation during mitosis [21–23]. Interestingly, emerin has also been found to be located at the outer nuclear membrane and peripheral Endoplasmic Reticulum (ER) with a direct interaction with the centrosome and microtubules [24]. Based on this additional role of emerin, one can expect *EMD* mutations to be found in other diseases related to centrosome.

Approximately 60% of EDMD cases seem to be caused by mutations in *EMD* [2]. Most are null mutations, which result in complete absence of emerin expression in nuclei. Furthermore, *FHL1*, the gene encoding for Four and a half LIM domains 1 (FHL1) proteins was discovered. In all tested mutated patients, the *FHL1* mutations were associated with severe reduction of FHL1 proteins and severe delay in myotube formation. Mutations in *FHL1* gene are responsible for about 10% of XL-EDMD [25]. EDMD-like syndrome can also be caused by rare mutations in several other genes, including *SYNE1* and *SYNE2* or *TMEM43* [26].

In the X-linked form of EDMD, female carriers are usually asymptomatic and unaffected. However, some rare cases of cardiac involvement in female carriers of *EMD* mutations have been described, in connection with unequal X-inactivation [27]. Very recently, a symptomatic female carrier of *EMD* mutation has been identified. This patient carries a heterozygous deletion (c.174_175delTT) that leads to a frameshift and the expression of a truncated protein. A mixed population of myoblasts, either emerin-positive or emerin-negative, was shown with a proliferative advantage for emerin-negative cells and a spontaneous differentiation phenotype for emerin-positive cells. The patient suffered from muscle weaknesses, myalgia, palpitations, and cardiac extrasystoles. These symptoms appeared between the late childhood and the early adulthood (from 12 to 23 years of age [28]).

Unfortunately, to date no curative treatment for EDMD is available. Only symptomatic treatments are available. They are based on orthopedic surgeries to limit contractures and scoliosis, the use of aids (walkers, wheelchairs) to preserve ambulation, and the management of cardiac features based on medication, pacemakers, and implantable cardioverter defibrillators (ICD). At the final stage of heart failure, heart transplantation can be a therapy to be considered (according to the benefit-risk balance).

### Mutations in the MAN1 gene (also called LEMD3)

Bone formation is affected in several LINC complex diseases. Increased bone density is the common symptom of osteopoikilosis, melorheostosis, and Buschke-Ollendorf Syndrome (BOS). BOS is a rare autosomal dominant disorder caused by LEMD3 loss-of-function, also known as dermatofibrosis lenticularis disseminate, and it is characterized by connective tissue nevi and osteopoikilosis. Its incidence is about 1/20,000 and the sex ratio is close to 1 [29].

This syndrome was initially described by Buschke and Ollendorf in 1928 [30]. The clinical features of BOS are inconstant with a great variability inside the same family: skin and skeletal symptoms may arise independently in affected family members. Osteopoikilosis, characterized by “spotted bones” (rounded or ovoid opacities on radiographic examination) is the consequence of osteosclerotic trabeculae. The dermatological manifestations can be divided in two different types: typical dermatofibrosis lenticularis disseminate (flesh-colored papules with symmetric distribution), or a “cobblestone” effect, made by the coalescence of papules. Melorheostosis is characterized by a floxing (rheos) hyperostosis of the cortex of trabecular bones. The association of skin lesions, even atypical, and a spotted bone pattern on the X-rays may lead to a genetic screening of the *LEMD3* gene (Fig. 4a) [31, 32].

MAN1, an integral protein of the inner nuclear membrane, influences transforming growth factor-β (TGF-β) signaling by directly interacting with R-Smads. Heterozygous *MAN1* loss-of-function mutations increase the level of TGF-β signaling in cells [33]. The bone sclerosis and overgrowth of connective tissue could be explained by enhanced cytokines signaling caused by partial loss of MAN1 from the INM [34, 35].

### Mutations in the LBR gene

The lamin B receptor (LBR) is an integral protein of the INM composed of several different domains. The N-terminal tail, which has a nucleoplasmic localization, can bind to B-type lamins, heterochromatin proteins, and DNA. This receptor has a hydrophobic domain, composed of several transmembrane segments with structural similarities to sterol reductases.

Homozygous mutations of the *LBR* gene lead to hydrops-ectopic calcification or Greenberg skeletal dysplasia, which is associated with lack of 3-beta-hydroxysterol delta-14 reductase activity. Thus, an abnormal sterol metabolite could be found in serum of patients suffering from Greenberg dysplasia: the cholesta-8,14-dien-3β-ol, signing for a sterol metabolism defect. Consequently, two hypotheses on pathogenic mechanisms have been proposed: either the disease is caused by metabolic defects, or the initial problem is an alteration in the nuclear structure leading to modifications in gene expression. A very recent study based on CRISPR-Cas9 technology showed that *LBR* point mutations are associated with a reduced sterol C14 reductase activity due and a lower affinity of LBR for NAPDH (Fig. 4a) [36].

Greenberg dysplasia is also called Hydrops, Ectopic calcification and Moth-eaten (HEM) skeletal dysplasia, which relates to the three main clinical features of the disease [37]. It begins during the second or third trimester of gestation, followed by fetal hydrops and death [37].

In a recent clinical report, a 15-year-old boy with an anadysplasia-like spondylometaphyseal dysplasia was described. This very mild skeletal dysplasia was caused by a double heterozygous mutation in the *LBR* gene [38]. This syndrome is characterized by anadysplasia-like features associated with spontaneous regression of associated radiographic skeletal abnormalities. However, a persistent, disproportionate and mild small stature was observed [38].

In contrast, most heterozygous mutations in *LBR* give a benign morphological granulocyte anomaly. This abnormality is visible in optical microscopy and leads to hyposegmentation and abnormal chromatin organization in nuclei, so called “Pelger-Huët anomaly”. However, *LBR* mutations that abolish sterol reductase activity could cause Greenberg skeletal dysplasia without Pelger-Huët anomaly [39]. A particular heterozygous missense mutation (p.Arg372Cys) has been reported to be associated with Reynolds syndrome. This auto-immune disorder is characterized by primary biliary cirrhosis, cutaneous systemic sclerosis but no Pelger-Huët anomaly [34].

These findings suggest that lamin B receptor has different domains supporting different functions of varying degrees of significance in different tissues [34]. Consequently, *LBR* mutations are involved in a large panel of diseases with a phenotypic heterogeneity of bone dysplasia caused by *LBR* mutations.

### Mutations in SUN genes

Five SUN proteins have been described in mammals: SUN1 and 2 are widely expressed, whereas SUN3, 4, and 5 are restricted to the testis [40].

Recent studies have suggested that *SUN1* and *SUN2*, genes that encode for SUN proteins, could be considered as modifier genes of a pre-existing disease [41]. It has been shown that mutations found only in *SUN* genes are not disease relevant [42, 43]. According to Meinke et al., *SUN* gene variants were identified in members of four families carrying *LMNA* or *EMD* mutations. Relatives who carry both mutations, a mutation in *SUN1* or *SUN2* associated with a mutation in *LMNA* or *EMD*, had a more severe disease than relatives who did not have a mutation in *SUN* genes. The presence of *SUN* variants could be an explanation to the great clinical heterogeneity of EDMD between relatives of the same family [42] (Fig. 4a).

In a study published by Chen et al., it was shown that the overexpression of *SUN1* gene is a critical pathogenic feature, observed in *Lmna* ^*−/−*^ and *Lmna* ^*Δ9–11*^ mice, and common to patients who suffer from Hutchinson-Gilford Progeria Syndrome (HGPS). Removal of *SUN1* gene in *Lmna* ^*−/−*^ and *Lmna* ^*Δ9–11*^ mice rescues their pathological phenotype and delays their premature death. At the cellular level, SUN1 proteins were mislocalized and accumulated in the Golgi apparatus, but it remains unclear if the accumulation of SUN1 in this organelle is responsible for a higher cell toxicity [44].

Mutations in *SUN1* or *SUN2* could affect the nuclear coupling to the cytoskeletal filament network [45]. These mutations could be responsible for abnormalities in nuclear movement and positioning in the cell. It was thought that mutations in SUN proteins cause abnormalities in nuclear-myotubule connection and prevent a correct myonuclei positioning. It has been admitted that nuclear dysmorphology is a feature that could be found in cells related to patients suffering from laminopathies. The consequences of this characteristic remain unclear [42].

In this first part, diseases caused by mutations in genes encoding for proteins of the inner nuclear membrane have been introduced. In the next sections of this review, diseases caused by mutations in genes coding for proteins which interact with this core will be developed: first, diseases caused by abnormalities of the nuclear lamina, located inside the nucleus, and then diseases caused by mutated proteins that are part of the outer nuclear membrane.

## Diseases caused by mutations affecting proteins of the nuclear lamina

### Mutations in the LMNA gene

Mutations in *LMNA,* coding for lamin A and C, are the cause of approximately a dozen inherited diseases, collectively called “laminopathies”, that were initially defined based on clinical signs and symptoms. Most are transmitted by dominant inheritance.

The laminopathies group includes: Autosomal Dominant form of EDMD (AD-EDMD or EDMD2), Autosomal Recessive form of EDMD (EDMD3), Dilated CardioMyopathy with Conduction Defect disease (DCM-CD), Congenital Muscular Dystrophy (L-CMD), Limb-Girdle Muscular Dystrophy 1B (LGMD1B), Dunningan-type Familial Partial Lipodystrophy (FPLD), atypical Werner syndrome, Charcot-Marie-Tooth syndrome 2B1 (CMT2B1), and Hutchinson-Gilford progeria syndrome (HGPS). Although highly variable, muscle defects are a frequently observed common clinical feature in these diseases. (Fig. 4b).

Muscle laminopathies such as EDMD2, DCM-CD, and LGMD1B, are characterized by joint contractures, primarily affecting the elbows, ankles and neck, progressive muscle weakness and wasting. The life-threatening symptom is cardiac conduction defects with dilated cardiomyopathy. These three diseases can be considered as a spectrum of the same pathology because clinical features overlap each other (Table 1) [46]. L-CMD due to *LMNA* mutations, associated with heart involvement and “heart-hand syndrome”, indicates that cardiomyopathy and congenital limb abnormalities are associated [47, 48].

**Table 1:** Clinical comparison of muscular phenotypes caused by LMNA mutations. Emery-Dreifuss Muscular Dystrophy (EDMD), Congenital Muscular Dystrophy (L-CMD), Limb-Girdle Muscular Dystrophy 1B (LGMD1B). Adapted from Helbling-Leclerc et al. [18] and Maggi et al. [113]

Age of onset	EDMD-2	LGMD1B	L-CMD
	2nd to 3rd decade	3rd to 4th decade	Congenital onset
Weakness	Scapulo-humero-peroneal distribution	Girdles: Pelvic and scapular, symmetrical	Axial or severe and diffuse
Contractures	Elbow +++	+	+ (spine, hips, knees and Achilles tendons)
Heart phenotype	Invariable with age, after skeletal muscle involvements Cardiac conduction defects +/− dilated cardiomyopathy	Invariable with age	Cardiac conduction abnormalities
Respiratory abnormalities	Rare	Rare	Very frequent
Loss of independent walking	Late but rare	Late but rare	Very frequent
Axial involvement	Frequent	Rare	Frequent
Scoliosis	Frequent	Rare	Frequent
Rigid Spine	Frequent	Rare	Frequent
Dysphagia	Very rare	Very rare	Very rare
Facial weakness	Rare	Very rare	Rare

FPLD is an autosomal dominant disease characterized by the loss of adipose tissue at the extremities, occurring at puberty. This disease has metabolic consequences such as insulin resistance, *diabetes mellitus*, hypertriglyceridaemia, and hepatic steatosis. Approximately 90% of *LMNA* mutations in FPLD are missense mutations in exon 8 of *LMNA* gene, causing an amino-acid substitution and leading to a modification of the surface charge of immunoglobulin-like fold domain of the C-terminal tail of lamins [49, 50]. This Single Nucleotide Variation (SNV) leads to an inhibition of adipogenic differentiation due to the deregulation of Sterol Response Element Binding Protein 1 (SREBP1), a crucial transcription factor for lipid metabolism and adipocyte differentiation [51].

CMT disease is the most common cause of inherited peripheral neuropathies with an estimated frequency of 1:2500. Electroneuromyographic examination distinguishes a myelinic form (CMT1) and an axonal form (CMT2) of the disease. Significant genetic heterogeneity is found in CMT, with 15 genes or loci for CMT2. CMT2B1 is an axonal form (CMT2) of the disease characterized by an autosomal recessive mode of inheritance and is a sensory neuropathy characterized by progressive muscular and sensory loss in the distal extremities with chronic distal weakness. CMT2B1 is associated with a homozygous amino-acid substitution (p.Arg298Cys) in the primary structure of the rod domain of lamin A and C. This substitution has only been found in affected families from a limited region of North Western Africa [52–55].

The last group of laminopathies implicates defects in multiple organs mimicking accelerated ageing [47] such as HGPS. Affected children appear normal at birth. During the first year of life, the symptoms of accelerated ageing occur: failure to thrive, delayed dentition, alopecia and sclerodermatous skin changes. Death occurs on average at age 13. The main death cause (90% of patients) is progressive atherosclerosis of the coronary and cerebrovascular arteries [56]. The main pathophysiological mechanism underlying progeria is an abnormal splicing of the *LMNA* primary transcript. Most HGPS patients carry a de novo point mutation within exon 11 of the *LMNA* gene (c.1824C > T, p.Gly608Gly). This mutation activates a cryptic splice site. This altered splicing leads to the deletion of 50 amino acids at C-terminal domain and to the accumulation of a truncated form of lamin A precursors, called progerin [57]. Recently, an atypical aggressive neonatal form of HGPS without progerin accumulation has been reported. The molecular exploration found an association of two de novo heterozygous point mutations in *LMNA*: c.163G > A, p.E55K and c.164A > G, p.E55G [58].

To date, several other rare inherited human diseases have been linked to *LMNA* mutations, such as an autosomal dominant form of lipoatrophy associated with diabetes, liver steatosis, dermatologic features (leukomelanodermic papules) and hypertrophic cardiomyopathy [59], A-type MandibuloAcral Dysplasia (MAD-A) [60, 61], atypical Werner’s syndrome [62, 63], a lethal form of restrictive dermopathy [64], and acrogeria syndrome [65]. Overlapping phenotypes between all these diseases have also been described, suggesting a clinical continuum between these clinical entities [66].

Despite the ubiquitous expression of lamins, most laminopathies involve highly tissue-specific phenotypes, often affecting skeletal and cardiac muscle. However, the mechanism of tissue-specificity remains unknown. Four hypotheses could be formulated. The first one, often called “structural hypothesis”, is based on the loss of structural functions of lamins A/C. Therefore, cells are more susceptible to suffer from mechanical damage. The second one proposes that lamins A/C interact with tissue-specific transcriptional regulators [67]. A third hypothesis proposes that *LMNA* mutation could cause impairments in muscle stem cell function. This hypothesis is based on the fact that, with the exception of peripheral neuropathy, which involves a cell type derived from ectoderm, the others primarily affect tissues are all of mesenchymal origin [68]. More recently, a last hypothesis has emerged based on the role of A-type lamins in RNA export through nuclear envelope budding. The accelerated aging linked with lamin C mutation in Drosophila, modeling *LMNA* mutations causing progeroid syndromes in humans, has been shown to be associated with RNA export defects causing loss of mitochondrial integrity [69].

Genetically, conversely to *EMD* mutations in XL-EDMD, *LMNA* mutations are mostly missense, causing amino-acid substitutions. Many other types of mutations are described: RNA splicing abnormalities, in-frame deletions, or haploinsufficiency caused by early chain termination. They are scattered all along the full length of the gene (except for HGPS). Nowadays, no clear correlation between genotypes and phenotypes has been established.

**Fig. 2:**
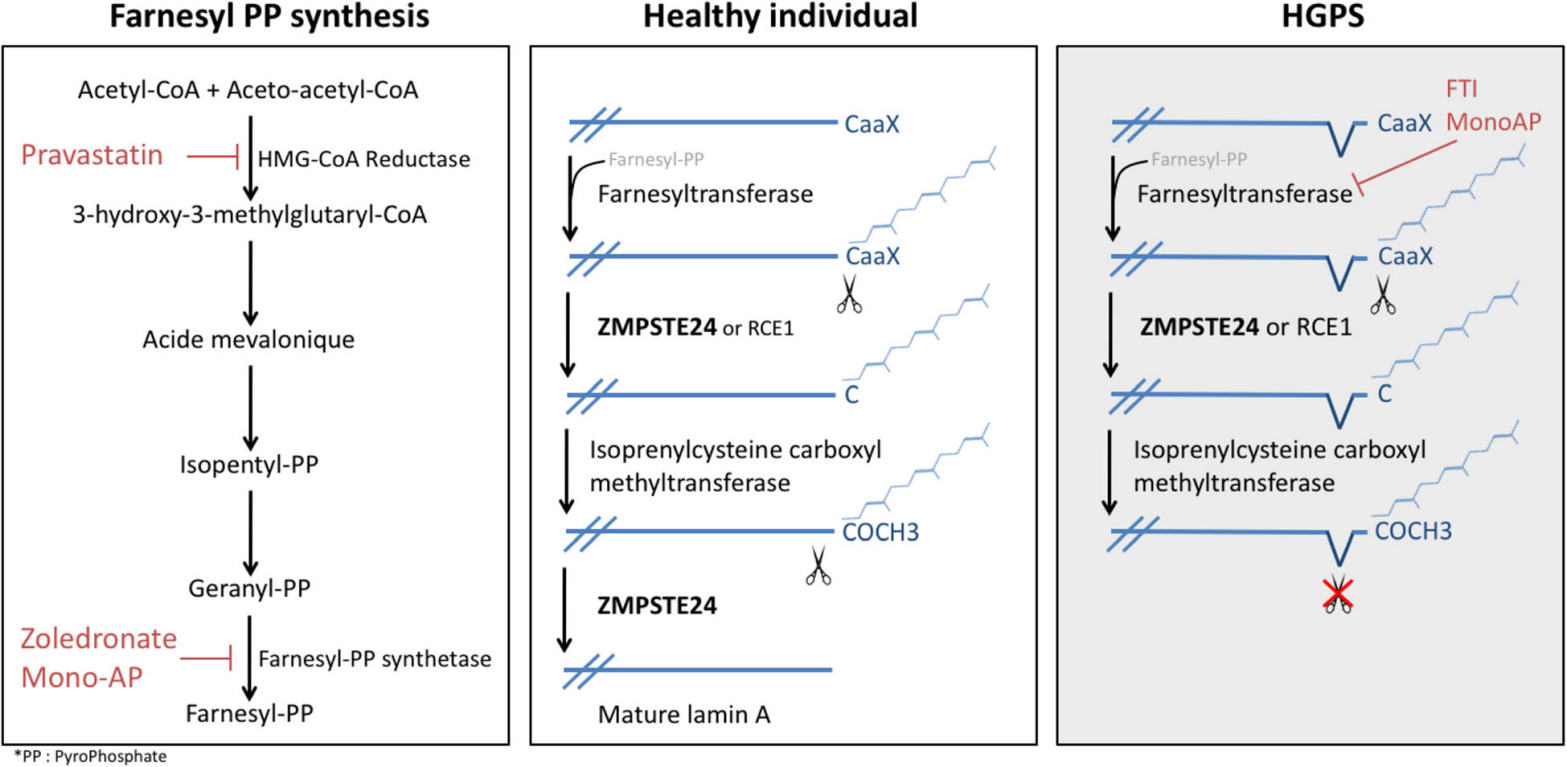
Prelamin A maturation process as a therapeutical target. Processing of prelamin A is a succession of enzymatic reactions that lead to a mature form of lamin A: The first step is the farnesylation of a cysteyl residue to obtain a farnesylated form of prelamin. Subsequently, a protease (ZMPSTE24 or RCE1) cleaves the aaX residues from the C-terminus tail. Finally, ZMPSTE24 protease cleaves the last 15 residues from the C-terminus to obtain mature prelamin A. In Hutchinson-Gilford Progeria Syndrome (HGPS) patient cells, the last cleavage by ZMPSTE24 does not take place leading to the abnormal accumulation of farnesylated lamin A. Potential therapeutic approaches are indicated in red: pravastatin, zoledronate, mono-aminopyrimidynes (mono-APs) and farnesyltransferase inhibitors (FTI)

To date, no curative treatment for laminopathies is available. Since the discovery of the molecular mechanisms underlying HGPS, different drugs have been thought to be helpful, based on their ability to interfere with the prenylation process of prelamin A. First, in 2005, the efficiency of FarnesylTransferase Inhibitors (FTI), such as lonafarnib, was assessed as a potential therapeutic treatment for HGPS with the hypothesis that the inhibition of progerin farnesylation would improve the nuclear phenotype by restoring a proper localization of lamin A [70]. Lonafarnib is currently in phase II trial for progeria [71]. The association of pravastine, a statin that inhibits HMG-CoA reductase, with zoledronate, an aminobisphosphonate that inhibits farnesyl pyrophosphate synthase (FPPS), is another potential therapeutic approach. This combination inhibits both farnesylation and geranylation of progerin and prelamin A. This combined approach, so called ZoPra, is associated with an improvement of the aging-like phenotypes of *Zmpste24* ^*−/−*^ mice recapitulating HGPS phenotypes such as growth retardation, loss of weight, lipodystrophy, hair loss, and bone defects [72] (Fig. 2). In 2013, a new type of FPPS inhibitor (N6-isopentenyladenosine) was discovered which improves nuclear shape abnormalities in fibroblasts from progeroid patients [73]. Adopting a different approach, an in vivo administration of temsirolimus, a rapamycin analog, was shown to be able to prevent deterioration of cardiac function. The mechanism underlying this effect seems to be an improvement of the autophagy that is found to be impaired in the heart of mutated mice. Temsirolimus was further shown to be able to partially rescue the cellular phenotype associated with HGPS [74, 75]. MG132, a protease inhibitor, is also found to improve HGPS cellular phenotype by induction of progerin degradation by macroautophagy and splicing regulation. Intramuscular injection of MG132 in skeletal muscle of Lmna^G609G/G609G^ mice locally reduces progerin levels. Protease inhibitors is another promising therapeutic class to treat HGPS patients [76]. Interestingly, although the molecular mechanisms of FTI, ZoPra and rapamycin are different, it was shown that the association of these 3 approaches lacks an additive effect [77]. Recently, a drug screening approach on iPS cell lines derived from HGPS patients identified mono-aminipyrimidines (mono-APs) as a family of molecules capable to restore HGPS cellular phenotype. Mono-APs act at two different levels: they inhibit both farnesyl pyrophosphate synthase and farnesyl transferase [78]. The same way, a drug testing approach on HGPS patient fibroblasts led to the discovery of a small molecule, called by the authors “remodelin”, able to improve nuclear architecture in these cells. Using mass spectrometry, they found that remodelin is an inhibitor of N-acetyltransferase 10 (NAT10). NAT10 inhibition rescues nuclear shape organization by microtubule reorganization. The supposed advantage of a remodelin-based treatment is a low toxicity of this drug at the cellular level [79].

Finally, a proof-of concept of exon-skipping therapy for laminopathies has been established: antisense oligonucleotides removing exon 5 of lamin A have been efficiently used in Human cells. It was further shown that lamin A/C-Δ5 normally localizes in murine *Lmna*-null primary murine embryonic fibroblasts and rescues the abnormal nuclear shapes commonly associated with laminopathies [80].

### Mutations in the LMNB1 and LMNB2 genes

Autosomal Dominant LeukoDystrophy (ADLD) is caused by a duplication of the *LMNB1* locus [81]. It is a rare genetic disorder, similar to chronic progressive multiple sclerosis, that leads to demyelination in the Central Nervous System (CNS). The age of onset is usually in the fourth or fifth decade of life, and it is slowly progressive and fatal. This syndrome is characterized by inconstant early autonomic abnormalities, pyramidal and cerebellar defects eventually associated with ataxia, cardiovascular, and skin defects. ADLD differs from multiple sclerosis as histological lesions display oligodendroglia preservation associated with subtotal demyelination and a decrease in astrogliosis. In addition, MRI finds diffuse subcortical white matter abnormalities [82] (Fig. 4b) .

Although no dominant-acting missense or loss of function mutations have been identified in B-type lamins, ADLD is associated with tandem duplications spanning *LMNB1* gene on chromosome 5q. The size of the duplication varies between families. This duplication is correlated with increased *LMNB1* mRNA levels and 2-fold increased level of proteins in white blood cells from patients. Consequently, ADLD could be caused by an effect of *LMNB1* over-expression on the transcriptional regulation of myelinogenesis genes [83]. More recently, studies based on proteomic and transcriptome assays have shown that lamin B1 overexpression causes a downregulation of proteolipid protein linked to a reduced occupancy of Yin Yang 1 (YY1) transcription factor at the promotor region of its gene [84]. Moreover, Lin ST et al. have shown that miR-23 is essential to regulate *LMNB1* expression and to have a normal oligodendroglia development [85]. Thus, a perspective field of study is to highlight the potential disappearance of the regulation mediated by miR-23 in affected families. Interestingly, an ADLD family without evidence of duplication or other mutation in *LMNB1* has been described. An array Comparative Genomic Hybridization (CGH) analysis led to identify a large (approximately 660 kb) heterozygous deletion 66 kb upstream of the *LMNB1* promotor. Lamin B1 overexpression was confirmed in a postmortem brain sample, showing that lamin B1 was increased in the frontal lobe. The deletion eliminates a genome topological domain boundary, allowing interactions between at least three forebrain-directed enhancers and the *LMNB1* promoter. This mechanism explains the cerebral localization of lamin B1 overexpression, myelin degeneration, and the ADLD phenotype [86].

Increased levels of lamin B1 have also been observed in lymphoblasts and fibroblasts from patients who suffer from ataxia telangiectasia (AT). AT is an autosomal recessive disorder characterized by cerebellar ataxia, telangiectasia, immune defects, and a predisposition to malignancy. As AT is caused by a mutation in the protein kinase ataxia telangiectasia mutated (ATM) that controls DNA damage response signaling, the link between AT and elevated levels of lamin B1 remains unsolved.

Conversely to the *LMNB1* gene, very few mutations in *LMNB2* have been associated with human diseases. The first reported heterozygous mutation of *LMNB2* gene is a case of acquired partial lipodystrophy, also called Barraquer-Simons syndrome [87]. More recently, the case of a consanguineous Palestinian Arab family showing an autosomal recessive progressive myoclonus epilepsy with early ataxia has been reported. This disease is a rare syndrome that could be associated with progressive antiepileptic drug resistance and cognitive decline. After linkage analysis and Sanger sequencing, a homozygous missense mutation (p.His157Tyr) in *LMNB2* gene segregating with the disease in this family was identified. The concerned amino-acid is located in a highly-conserved region of the protein (alpha-helical rod region). In vitro studies suggest that the mutation could affect the correct assembly of the protein. This mis-assembly could cause an abnormal neuronal migration that leads to the epilepsy and early ataxia syndrome (Fig. 4b) [88].

### Mutations in the LAP2a and BANF1 genes

The lamina-associated polypeptide 2α (LAP2a) is a LEM (LAP2-Emerin-MAN1) domain protein. This domain is a structural motif of about 150 N-terminal amino-acids that binds to BAF. The latter is encoded by *BANF1*, mediates the interaction with chromatin, and plays a central role in nuclear organization and nuclear envelope assembly. Unlike the other LAP2 isoforms, LAP2a is not anchored in the INM but is uniformly distributed in the nucleoplasm. The protein is composed of 3 main domains: the first binds to A-type lamins, the second is a chromosome association domain, and the third mediates binding to BAF. Thanks to these regions, LAP2a plays a major role in the regulation and stabilization of the lamin A/C nucleoplasmic pool [89].

In 2005, a *LAP2a* mutation associated with dilated cardiomyopathy (DCM) characterized by an autosomal-dominant mode of inheritance was described. The clinical features are very close to those found in DCM caused by *LMNA* mutations: age of onset between 20 and 30 years, decrease in left ventricular ejection fraction but no cardiac conduction abnormality was observed. The identified mutation is a heterozygous substitution (p.Arg690Cys) affecting a residue located in the C-terminal domain implicated in the binding to lamin A/C. Consequently, the LAP2a mutated protein shows a lower affinity for prelamin A. However, the precise pathophysiological mechanism that leads to DCM remains unclear (Fig. 4b) [90].

In 2011, a *BANF1* homozygous mutation (p.Ala12Thr) reported in 2 Spanish families, found by exome sequencing method, was described as the cause of a progeroid syndrome, with a phenotype extremely close to that of HGPS. The syndrome, called Nestor-Guillermo Progeria Syndrome (NGPS), partially phenocopies HGPS but with abnormal clinical features: tardive age of onset, taller patients, presence of eyebrows and eyelashes, absence or complete loss of scalp hair, a severe osteolysis and the absence of cardiovascular or metabolic defects. However, NGPS and HGPS patients share common symptoms: aged appearance, growth retardation, thin limbs, stiff joints, and loss of subcutaneaous fat. Moreover, no mutation in *LMNA* or *ZMPSTE24* has been found. This mutation could affect the stability of the protein as no decrease in mRNA expression level was found. The *BANF1* mutation leads to an abnormal distribution of nuclear lamina components and leads to nuclear abnormalities. Finally, children who suffer from this syndrome do not have an increased risk of acute myocardial infarction, cerebrovascular accidents and *diabetes mellitu*s [91, 92] (Fig. 4b).

## Diseases caused by mutations affecting proteins of the outer nuclear membrane

### Mutations in the ZMPSTE24 gene

ZMPSTE24, also known as Farnesylated-protein Converting Enzyme 1 (FACE-1), is a zinc metalloprotease that plays a central role in the maturation of prelamin A to mature lamin A. Lamin A and B must go through a cascade of C-terminal post-translational modifications. The first step is a farnesylation, that is undertaken by a farnesyltransferase, of a cysteine included in a CAAX pattern (where A is an aliphatic residue and X stands for any residue). ZMPSTE24 or RCE1 proteases will first cleave the AAX residues from the C-terminus of prelamin A, then the farnesylated cysteine will be carboxymethylated by an isoprenylcysteine carboxymethyltransferase. Subsequently, ZMPSTE24 cleaves an additional 15 residues from the C terminus of prelamin A, leading to mature lamin A that does not retain the hydrophobic modifications [93–95]. B-type lamin will not undergo this final cleavage and will keep the modified tail (Fig. 2).

**Fig. 3:**
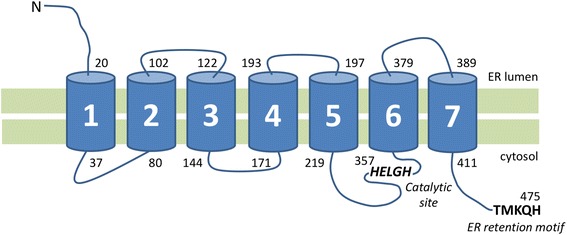
Predicted structure of ZMPSTE24 protease. ZMPSTE24 is a transmembrane protein located in the Outer Nuclear Membrane composed of seven hydrophobic domains (1 to 7, a catalytic domain (HELGH residues), and an endoplasmic reticulum retention motif (TMKQH residues)

ZMPSTE24 is a membrane-associated enzyme with 7 transmembrane segments (Fig. 3) located in the ER membrane. As previously described for emerin, the localization of this protein is not clear and an additional localization at the inner nuclear membrane has been proposed. The enzyme contains a consensus zinc metalloprotease motif, located in the cell cytosol, that is a HEXXH catalytic site [95].

**Fig. 4:**
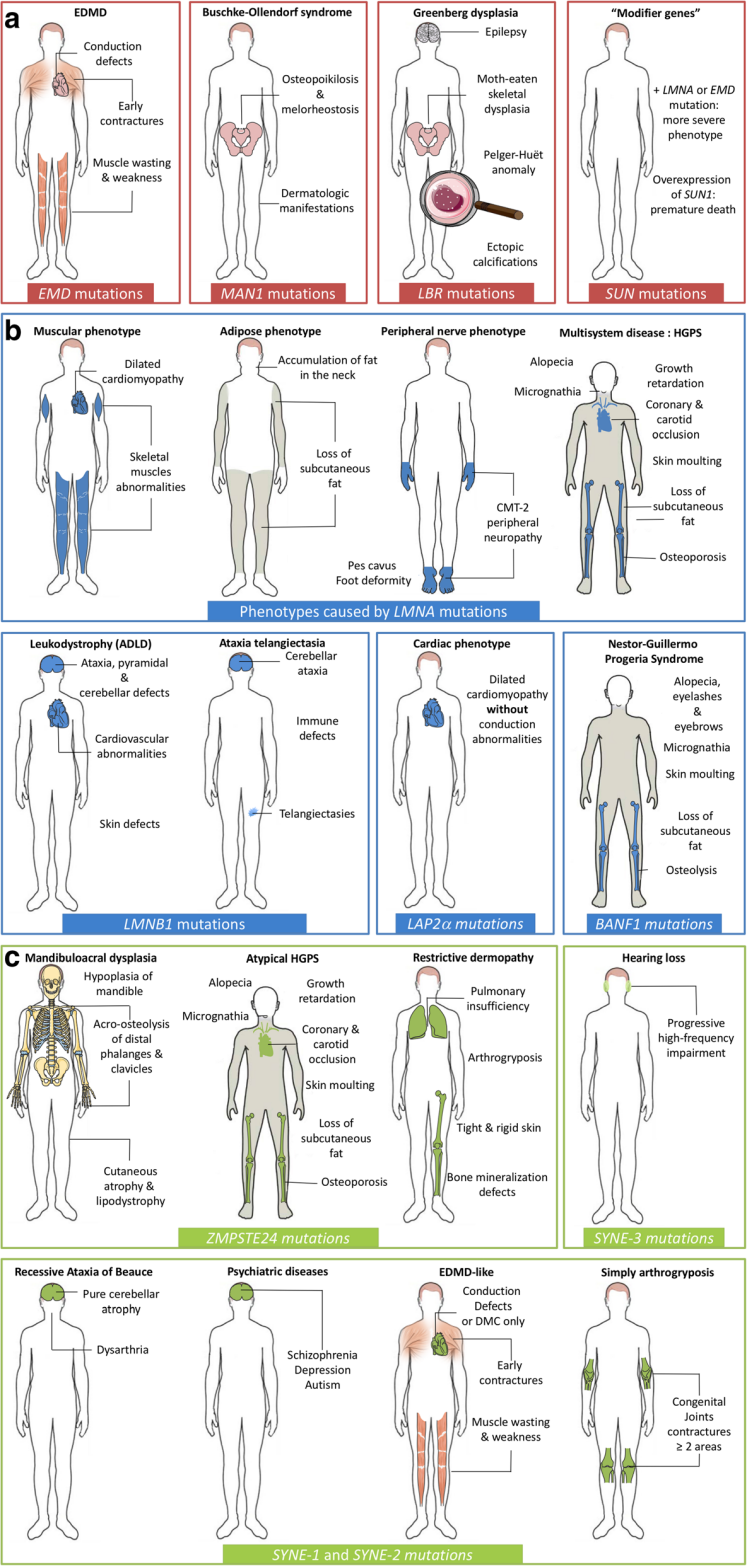
Summary of all known human diseases caused by mutations in genes coding for nuclear envelope components. The diversity of phenotypes induced by mutations in genes encoding nuclear envelope components, as well as the tissues affected by these, is illustrated and organized according to the localization of the mutated protein: (**a**) inner nuclear membrane, (**b**) nuclear lamina, and (**c**) outer nuclear membrane components. EDMD: Emery-Dreifuss Muscular Dystrophy, HGPS: Hutchinson-Gilford Progeria Syndrome, CMT: Charcot-Marie-Tooth Disease, ADLD: Autosomal Dominant Leukodystrophy

Homozygous mutations in the *ZMPSTE24* gene result in progeroid syndromes due to the accumulation of an farnesylated form of prelamin A (Fig. 4c).

Mandibuloacral dysplasia associated with B-type lipodystrophy or MAD-B (also known as “atypical HGPS”) is the less severe form of diseases caused by *ZMPSTE24* mutations. Clinically, the main features are skeletal abnormalities including hypoplasia of the mandible and clavicles, acro-osteolysis of distal phalanges, cutaneous atrophy, and lipodystrophy. In typical MAD-B, lipodystrophy is generalized. The age of onset is usually during the first year, the median age of death is approximately 30 years (Fig. 4c).

The lethal neonatal Restrictive Dermopathy (RD) is the most severe pathology caused by *ZMPSTE24* mutations, which is a rare and extremely severe congenital genodermatosis (inherited genetic skin condition). The first symptom consists of intrauterine growth retardation, associated with fetal hypokinesia deformation sequence (characterized by a reduction of fetal movements). The main clinical feature is a tight and rigid skin: rare or absent eyelashes, erosion at flexure sites, hyperkeratosis, microstomia (reduction in the size of the oral aperture) characterized by a particular position in letter ‘O’, and a small and pinched nose. It is associated with prominent superficial vessels, bone mineralization defects, dysplastic clavicles, arthrogryposis (congenital joint contractures) and pulmonary insufficiency that is usually the cause of early neonatal death [96]. According to recent studies, the typical RD phenotype seems to be due to *ZMPSTE24* null mutations and complete loss-of-function whereas less severe phenotypes could be associated with *ZMPSTE24* haploinsufficiency or *LMNA* mutations (often called “RD-like phenotypes”) (Fig. 4c) [97, 98].

More recently, the phenotype of a patient carrying a heterozygous point mutation in *ZMPSTE24* gene has been reported. The patient suffers from a severe metabolic syndrome (partial lipodystophy, hypertriglyceridemia, early onset type 2 diabetes mellitus, android obesity without subcutaneous lipoatrophy) associated with dilated cardiomyopathy, acanthosis nigricans, liver steatosis [99]. Unfortunately, no segregation study could be performed and the link between the phenotype and the mutation remains unsure.

Mutations concern mostly a stretch of thymines in exon 9 of *ZMPSTE24*: the hotspot is c.1085dupT or p.Leu362Phefs*19. This mutation leads to a frameshift. The consequence is the lack of the last transmembrane domain and the ER retention signal. Ultimately, this duplication leads to the complete loss of ZMPSTE24 enzymatic activity due to the complete absence of protein, confirmed by Western blot analysis. According to a recent study, this mutation has been found in 59.1% of all others mutations in *ZMPSTE24* associated with the RD phenotype, and in 18.8% associated with MAD-B or overlapping HGPS/MAD syndrome [97].

All mutations found by molecular testing in patients with RD or MAD-B are homozygous or compound heterozygous. Thus, correlated with the recessive inheritance of the disease, heterozygous mutations found in patients’ relatives are apparently not deleterious. Always according to the same recent study published by Navarro et al., all mutations associated with the RD phenotype have been shown to be null mutations, such as the common c.1085dupT mutation. Practically, all types of null mutations could be found: nonsense mutations, insertions and deletions with frameshift, frameshifts and premature stop codons caused by splice sites mutations. Conversely, all patients with no RD phenotypes are compound heterozygous in which a null mutation is found in the first allele associated with a missense mutation in the second [93, 97].

### Mutations in SYNE genes

Nesprins have been characterized during the last 10 years as spectrin-repeat proteins. Nesprin-1 and nesprin-2 are encoded by two independent genes, *SYNE1* and *SYNE2*, but multiple protein isoforms are generated by alternative initiation and splicing.

### Nesprin-1 and Nesprin-2

*SYNE1*, coding for nesprin-1, has recently been shown as neurodegenerative diseases causative gene. Mutations in *SYNE1* have been identified in French and Canadian families. The phenotype associated with these mutations is Autosomal Recessive Cerebellar Ataxia type 1 (ARCA1) also called “recessive ataxia of Beauce” [100]. In this case, the disease is characterized by a slow progression and a late-onset. Moreover, the phenotype is uniform between patients: diffuse pure cerebellar atrophy, dysarthria but no muscular features. In most cases, non-sense mutations and intronic mutations causing premature termination were found in these patients [101]. On another hand, mutations in *SYNE1* found in Japanese patients correlated with SpinoCerebellar ataxia, Autosomal Recessive type 8 (SCAR8) associated with motor neuron disease. In these patients, the phenotype begins as an amyotrophic lateral sclerosis of juvenile onset associated with progressive muscular atrophy. Only later, they develop features related to cerebellar ataxia: inability to coordinate movements and dysarthria [102]. A recent report of two siblings carrying *SYNE1* premature termination codon (PTC) mutation with a misdiagnosis for multiple sclerosis for more than a decade has been published. This paper highlights the heterogeneity of the clinical presentation of ARCA1 with potential white matter abnormalities on MRI showing that ARCA1 is not a pure cerebellar degeneration [103].

It has since been recognized that *SYNE1* could be involved in schizophrenia, depression and autism. For example, mutation in *SYNE1* is considered as a risk factor for schizophrenia. Moreover, recent whole-exome sequencing studies have identified *SYNE1* as an autism spectrum disorder (ASD) candidate gene [104] and more particularly, the homozygous p.Leu3206Met mutation**.** Finally, correlations between mutations in *SYNE1* with bipolar disorder or depression have been found [105].

Concerning their roles in musculoskeletal diseases, mutations in *SYNE1* and *SYNE2* are also associated with AD-EDMD and EDMD-like phenotypes. In a study published in 2007, the analysis of *SYNE1* and *SYNE2* genomic sequences were performed in 190 patients who suffer from EDMD or EDMD-like phenotype and for whom no mutation in *LMNA* or *EMD* were found. Six unique DNA variants, absent from a control population, were identified. Segregation analysis in affected families was performed and the segregation pattern was compatible with an autosomal-dominant inheritance. These mutations lead to nuclear defects and mislocalization of nesprin, and lamin from the nuclear envelope in fibroblasts derived from patients [106]. Mutations in *SYNE1* are also associated with DCM with conduction system defects. Very recently, other point mutations have been reported to be associated with a DCM phenotype. Fibroblasts from one patients (carrying the p.Arg374His mutation) were isolated and an increased expression of nesprin-1 (four-fold) and lamin A/C (three-fold) without mislocalization were found [107, 108]. These data are evidence that LINC complex perturbations in general are susceptible to cause skeletal and heart diseases. Thanks to genotype to phenotype correlation studies, it has been shown that mutations in the C-terminal region of nesprin 1 and 2 are associated with muscular disorders whereas mutation in the N-terminal regions are linked to ataxia [108, 109].

Mutations in *SYNE1* are responsible for Arthrogryposis Multiplex Congenital (AMC) or simply arthrogryposis. It is a group of non-progressive diseases characterized by congenital joint contractures, in two or more areas of the body, caused by reduced fetal movements. Prevalence varies from 1/12.000 to 1/3.000 newborns (Fig. 4c) [110].

Finally, recently, a new role of nesprin-1 in the formation of striated F-actin-based filaments has been described. Such filaments, so called “railroad tracks” by the authors, take place in the muscle from the nucleus to the synaptic membrane. Interestingly, the absence of nesprin-1 is associated with a mislocalization of mRNA at postsynaptic sites causing an impaired synaptic maturation [111]. This emerging role suggests that *SYNE1* mutations could be discovered in other neuromuscular junction diseases.

### Nesprin-3

Available data concerning the role of nesprin-3, encoded by *SYNE3*, are more limited. To date, no disease associated with mutations in *SYNE3* has been reported. In vitro studies based on inactivation using short-interferent-RNA have recently established that nesprin-3 plays a central role in cytoskeletal perinuclear organization, embryonic development, and preservation of tissue integrity suggesting it could also be affected in developmental diseases [101].

### Nesprin-4

Nesprin-4, which is encoded by *SYNE4*, is surprisingly, and in contrast with the ubiquitous expression of the others nesprins, exclusively produced by secretory epithelia and mechanosensory cochlea hair cells. Mutations in *SYNE4* are involved in hearing loss characterized by hereditary and progressive high-frequency impairment. A loss-of-function mutation based on a frameshift caused by a two nucleotides deletion (c.228delAT) has been recently identified [112].

## Conclusions

Several new and sometimes unexpected functions have recently been attributed to the cell nucleus, and more particularly to the nuclear envelope. The nuclear envelope not only ensures the integrity of the cell nucleus, but is also implicated in mechanotransduction signaling by sensing and relaying the cytoskeleton tension it interacts with. The same way, the interactions between chromatin and the inner nuclear membrane and nuclear lamina are not only crucial to coordinate and regulate gene expression, but can also be organized to improve light detection in photoreceptor rod cells of nocturnal animals [86, 87]. The nuclear envelope is not anymore considered as a simple lipid double membrane separating the cytoplasm and nucleoplasm but is recognized as a complex interface organizing both the genome and the cytoskeleton. This dual role is mediated by multiprotein complexes establishing physical interactions between the nuclear envelope and the sarcolemmal proteins on one side and the nuclear lamina on the other side.

In recent years, several components and interactors of the LINC complex have been identified either covering the inner surface of the nucleus, or located in the inner or outer nuclear membrane. In parallel, mutations in genes encoding for nuclear envelope components have been associated with rare human diseases affecting numerous different tissues (Fig. 4). Despite several databases and studies listing hundreds of mutations, no clear correlation between a given genotype and its affected tissues or disease has been established. For example, mutations in emerin, an inner nuclear membrane protein, lamins A/C, the main component of the nuclear lamina, or FHL1, a transcription factor, can all lead to EDMD affecting both the skeletal muscle and heart. However, mutations in lamins A/C can also be responsible for pathologies affecting the peripheral nerve or adipose tissue making the molecular diagnosis of envelopathies particularly difficult. Moreover, the identification of variants in the *SUN* gene modulating the severity of an existing disease due to a mutation in another component of the LINC complex underlines the importance of looking for mutations or variants in all currently known genes encoding LINC components in patients potentially affected by a nuclear envelopathy.

One can expect that the routine use of Next Generation Sequencing (NGS) tools in diagnosis laboratories will accelerate this discovery process and will help to better understand the pathophysiological mechanisms underlying rare pathologies without molecular explanation.

## Abbreviations

ATM: Ataxia Telangiectasia Mutated
BAF: Barrier of Autointegration Factor
CGH: Comparative Genomic Hybridization
CK: Creatine Kinase
CMT: Charcot-Marie-Tooth
CRISPR: Clustered Regularly Interspaced Short Palindromic Repeats
DCM-CD: Dilated CardioMyopathy with Conduction Defect
EDMD: Emery-Dreifuss Muscular Dystrophy
FACE-1: FArnesylated-protein Converting Enzyme 1
FHL1: Four and Half LIM domains 1
HGPS: Hutchinson-Gilford Progeria Syndrome
INM: Inner Nuclear Membrane
LAP2: Lamin-Associated Polypeptide
LBR: Lamin B Receptor
LGMD1B: Limb-Girdle Musuclar Dystrophy 1B
LINC: LInker of Nucleoskeleton to Cytoskeleton
MAD-B: Mandibuloacral Dysplasia B
MRI: Magnetic Resonance Imaging
NGPS: Néstor-Guillermo Progeria Syndrome
ONM: Outer Nuclear Membrane
RD: Restrictive Dermopathy
SNV: Single Nucleotide Variation
YY1: Yin Yang 1
